# Antidiabetic activities of aqueous ethanol and n-butanol fraction of *Moringa stenopetala* leaves in streptozotocin-induced diabetic rats

**DOI:** 10.1186/s12906-015-0779-0

**Published:** 2015-07-18

**Authors:** Alemayehu Toma, Eyasu Makonnen, Yelamtsehay Mekonnen, Asfaw Debella, Sirichai Adisakwattana

**Affiliations:** Pharmacology Department, School of Medicine, Addis Ababa University, Addis Ababa, Ethiopia; Pharmacology Unit, School of Medicine, Hawassa University, P.o. box 1560, Hawassa, Ethiopia; Department of Biology, College of Natural and Computational Sciences, Addis Ababa University, Addis Ababa, Ethiopia; Directorate of Traditional and Modern Drug Research, Ethiopian Public Health Institute (EPHI, Addis Ababa, Ethiopia; Department of Nutrition and Dietetics, Faculty of Allied Health Sciences, Chulalongkorn University, Pathumwan, 10330 Bangkok Thailand

**Keywords:** *Antihyperglycemic*, *Antihyperlipidemic*, *Streptozotocin*, *Moringa stenopetala*, Histopathology

## Abstract

**Background:**

*Moringa stenopetala* has been used in traditional health systems to treat diabetes mellitus. The aim of this study was to investigate the antidiabetic activity of aqueous ethanol and n-butanol fraction of *Moringa stenopetala* leaves in streptozotocin (STZ) induced diabetic rats.

**Methods:**

The aqueous ethanol extract and n-butanol fraction of *Moringa stenopetala* leaves hydroalcoholic (500 mg/kg body weight) and metformin (150 mg/kg body weight) were administered to diabetic rats. Blood glucose, lipid profiles, liver and kidney function were examined after 14 days of experiment. Histopathological profile of the pancreas was also observed in diabetic rats at the end of study. An oral sucrose challenge test was also carried out to assess the post prandial effect of the extract.

**Results:**

Oral administration of the aqueous ethanol and n-butanol extracts of *Moringa stenopetala* leaves (500 mg/kg body weight) and metformin (150 mg/kg) significantly reduced blood glucose level (P < 0.05), improved serum lipid profiles, liver enzymes and kidney functions in diabetic rats after 14 days. The extracts also improved damage of islet of Langerhan’s in diabetic rats. The plant material reduced the post-prandial glucose level (P < 0.001) at the dose of 750 mg/kg.

**Conclusion:**

These findings revealed that both the aqueous ethanol and n-butanol extracts of *Moringa stenopetala* leaves possess antihyperglycemic and antihyperlipidemic properties, and alleviate STZ-induced pancreatic damage in diabetic rats. The beneficial effects of plant material in inhibition of diabetes-induced complications are being investigated.

## Background

Worldwide, the number of people with diabetes and pre-diabetes is exponentially increasing mainly due to aging, urbanization, unhealthy eating habits, increasing prevalence of obesity and lack of physical activity [1]. Diabetes mellitus is a leading cause of morbidity and mortality worldwide, with an estimated 382 million adults being affected and 5.1 million people killed in 2013. The prevalence is expected to be 592 million in 2035, with the greatest increases expected in low- and middle-income developing countries of the African, Asian, and South American regions. At present, 80 % of the world’s populations with diabetes live in low- and middle income countries [2]. Diabetes is also associated with a host of life threatening and potentially disabling macro- and micro-vascular complications [3]. Hence, there is a much larger burden in the form of loss of productivity as a result of restricted daily activity which results in high economic costs. Within the increasing interest to improve a quality and healthy life, herbal medicines with blood glucose lowering effects have been used for alternative treatment of type 2 diabetes mellitus.

*Moringa stenopetala (Baker f) Cufodontis* belongs to family Moringaceae is commonly grown in Southern parts of Ethiopia [4]. The leaves of *Moringa stenopetala* are cooked and eaten as vegetables and the leaves and roots are used to treat malaria, diabetes, asthma, repelled placenta, hypertension and gastrointestinal problems [5, 6]. It has been reported that *Moringa stenopetala* leaves and roots showed antitrypanosomal activity [7]. The antispasmodic effects of the leaves on smooth muscle tissues and antibiotic properties of the seeds [5, 8] have also been reported. Hypoglycemic activity has been demonstrated in the crude aqueous extract of the leaves in normal mice [9]. The crude aqueous/ethanol extract and fractions of the leaves of *Moringa stenopetala* have been reported to have both hypoglycemic and antihyperglycemic effects [10, 11]. Moreover, chronic administration of the n-butanol fraction of ethanol extract of *Moringa stenopetala* leaves in alloxan-induced diabetic mice showed antihyperglycemic and antihyperlipedimic effects with wide margins of safety, indicating its potential for long term management of diabetes and dysilipidemia through enzyme inhibition mechanisms [12–14]. The aim of the present study was to investigate the pancreatoprotective effect of the aqueous ethanol and n-butanol fraction of *Moringa stenopetala* leaves on streptozotocin (STZ)-induced diabetic rats on blood glucose, serum lipid levels, hepatic enzymes and kidney function.

## Methods

### Chemicals

Streptozotocin was purchased from (Sigma Aldrich, Germany), Metformin was purchased from (Sanofi winthrop industrie, France), SensoCard glucometer and strip was purchased from(77 Electronike Kft, Hungary). All others chemicals used were of analytical grade.

### Collections and preparation of plant materials

The leaves of *Moringa stenopetala* were collected from Gamo Gofa Zone, South Nation’s Nationalities Peoples Region, and 520 kilometers south of Addis Ababa. After collection, the plant was identified and authenticated by a taxonomist, and deposited in the herbarium of Ethiopian Public Health Institute (EPHI) with a voucher number AL-001. It was then dried under shade and crushed to powder for extraction.

### Preparation of plant material extract

The powdered leaves (1.2 Kg) were extracted by percolation using 70 %( v/v) ethanol, and the mixture was then filtered using Whatmann filter paper no. 1. The extract was dried by evaporation using rotary vaporizers under reduced pressure at a temperature of 40-45 °C. The residue filtrate obtained was then dried by steam bath at 40 °C and kept in a refrigerator at 8 °C for experimental usage. The yield of the aqueous ethanol extract was 20.1 % in weight by weight (w/w).

### Solvent fractionation of the total ethanol extract

The procedure for solvent-solvent separation was adopted from Ranjan [15] with minor modifications. Ten percent (w/v) of ethanol extract of the plant was prepared with mild hot distilled water. The dissolved aqueous ethanol extract was separated in a separatory funnel with 50 ml of n-hexane (3 times), and followed by 50 ml of dichloromethane extraction (3 times) and 50 ml of n-butanol extraction (3 times) successively until the extracting solvent became colorless. After completing the separation process, the solvents were removed by a rotary evaporator. The separated n-butanol separation were dried by steam bath at 40 °C and kept in the refrigerator for the experiments. The percentage yield of n- butanol was 7.8 (w/w).

### Animals

The Swiss albino rats weighing 180-250 g were obtained from the animal department of Ethiopian Public Health Institute/EPHI/. They were kept under standard conditions (at a temperature of 22 ± 2 °C, and with 12 hr light/ 12 hr dark cycle) and provided with free access to standard pellet laboratory diet and water *ad libitum*. The experimental protocol was approved by the Institutional review board (IRB) of Addis Ababa University, School of Medicine with protocol number 001/13/pharma.

### Induction of experimental diabetes

Six rats were randomly selected as normal controls. Diabetic rats were induced by streptozotocin (STZ) 40 mg/kg body weight, intraperitonially (IP). STZ was dissolved in citrate buffer solution (0.01 M, pH = 4.5). All the animals had free access to water and pellet diet after thirty minutes of administration. Five days thereafter the fasting blood glucose levels of rats were determined using glucose oxidase method with glucose analyzer. A blood glucose level greater than 126 mg/dl was considered to be diabetes mellitus (DM). Streptozotocin induced diabetic rats were selected and divided in four groups; negative control, positive control, and test groups.

### Sucrose tolerance test

The normal mice were divided into five groups and each group contained six animals. The control group was fed with distilled water. Other groups were fed orally with three different doses of aqueous ethanol extract of *Moringa stenopetala* leaves (250, 500 and 750 mg/kg). For the last group, acarbose (3 mg/kg) was used as a positive control. All treatments were administered to the mice 5 min before loading sucrose (3 g/kg). Blood samples were collected from a tail vein at 0, 30, 60, 90 and 180 min. The blood glucose levels were determined by glucose oxidase method.

### Study on effect of aqueous ethanol extract and n-butanol fraction on blood glucose levels

Diabetic rats were administered with 500 mg/kg body weight of ethanol extract and butanol fraction daily for 14 days via oral gavage. The diabetic and normal rats were given 10 ml/kg of body weight of normal saline via oral gavage and positive control rats were given 150 mg/kg of metformin via oral gavage. On days 0, 7, and 14 the blood samples were collected from tail vein following overnight fasting and blood glucose levels were measured. Body weight of each rat was determined at the same time.

### Assay of serum lipid level, hepatic enzymes and renal function tests

On day 15, the rats were fasted overnight, blood samples were collected in a sterile tube by cardiac puncture under ether anesthesia and left to stand at room temperature for 2 h, then centrifuged at 1500 × g for 15 min at 4 °C. The supernatant was immediately separated from the pellet to prepare serum samples in order to determine the level of triglyceride (TG), total cholesterol (TC), high density lipoprotein (HDL), aspretate aminotransferase (AST), alanine aminotransferase (ALT), alkaline phosphatase (ALP), gamma gluthathione transferase (GGT), bilirubin total (BLT), albumin, urea and creatinine using automated chemistry analyzer (humostar 80, Germany).

### Histological sample preparation

After sacrifice, the body of pancreas was dissected, collected and fixed in 10 % neutral buffered formalin. The samples were processed in graded series of alcohol and embedded in paraffin wax, sectioned at 5 μm and stained with hematoxylin and eosin for histological examination.

### Statistical analysis

All the values of body weight, fasting blood sugar and serum biochemical parameters were expressed as mean ± standard error of mean (SEM). The values were analyzed by one-way analysis of variance (ANOVA) followed by the Tukey’s post hoc test. The level of statistical significance was set at P < 0.05. Analysis was performed using SPSS software package Version 20.0.

## Results

### Effect of *Moringa stenopetala* leaf extract on postprandial glycemia in non-diabetic mice

A single oral administration of Moringa stenopetala extract was given to non-diabetic rats to confirm its antihyperglycemic activity by oral sucrose tolerance test. The results showed that blood glucose level reached the peak at 30 min after sucrose administration in all groups (Table 1). In addition, the extract at a dose of 250 mg/kg, 500 mg/kg and 750 mg/kg significantly reduced a raised blood glucose.The positive control, acarbose, significantly reduced blood glucose after 30, 60 and 120 min of administration relative to non-treated control rats (Table 1).

**Table 1:** Sucrose challenge test of hydroalcoholic extract of Moringa stenopetala in mice

	Blood glucose(mg/dl) level in different time intervals (minutes)				
Groups	0	30	60	120	180
Control	203.2±1.75	301.5±0.79	274.2±1.01	243.1±1.11	195.6±0.53
250 mg/kg MS extract	192.3±1.12	253.3±0.98*	226.2±0.78*	195.6±0.67*	152.3±0.78*
500 mg/kg MS extract	189.3±1.23	249.3±0.91*	233.2±1.13*	189.3±0.57**	140.8±0.45**
750 mg/kg MS extract	193.8±1.79	255.3±2.11*	226.1±1.10*	182.8±0.58**	133.4±0.23***
3 mg/kg Acrabose	192.8±0.74	223.6±1.71***	171.0±0.79***	155.6±1.33***	124.8±0.37***

Results are expressed in Mean ± S.E.M, n = 6

*when p < 0.05

**when p < 0.01

***when p < 0.001 verses control

### Effects of *Moringa stenopetala* leaf extract on blood glucose level in normal and diabetic rats

Blood glucose levels were measured once weekly in diabetic and normal rats given *Moringa stenopetala* extract after the induction of diabetes. The results are summarized in Table 2. Before induction of diabetes, there was no significant difference in blood glucose levels between diabetic and non diabetic control groups (p > 0.05). After induction, diabetic rats showed significant differences in blood glucose levels compared to normal rats (p < 0.05). The groups treated with the aqueous ethanol extract and n-butanol fraction of *Moringa stenopetala* leaves showed significant reduction of blood glucose level on day 7 and 14 after administration. In addition, the lowering blood glucose level in diabetic rats was also observed throughout the 14 days of study when metformin was administered.

**Table 2:** Effects of aqueous ethanol and n-butanol fraction of *Moringa stenopetala* leaves on blood glucose level in diabetic rats

Fasting blood glucose level (mg/dl)				
Treatment Groups	Before Induction	Day 0	Day 7	Day 14
Normal control	114.00±2.11	109.67±3.39^a^	104.67±2.79^a^	104.00±3.45^a^
Diabetic Control	115.10±1.75	145.17±1.05^b^	140.50±2.29^b^	136.10±2.52^b^
DC + 500 mg/Kg ethanol extract of MS	111.67±1.19	141.56±1.50^b^	123.83±1.99^ab^	120.33±2.23^ab^
DC + 500 mg/Kg butanol fraction of MS	115.17±2.15	141.56±1.45^b^	129.67±2.40^ab^	123.00±1.94^ab^
DC + I50 mg/Kg Metformin	112.33±2.09	142.17±1.92^b^	124.67±2.81^ab^	117.83±3.05^ab^

Results are expressed in Mean ± S.E.M, n = 6

^a^when p < 0.05 verses Diabetic control

^b^when p < 0.05 verses normal control

### Effect of *Moringa stenopetala* leaves extract on body weight

The body weight of diabetic and normal rats given *Moringa stenopetala* extract after the induction of diabetes was measured in the same rats as in the previous result (Table 3). There were no significant differences in the initial body weights among the groups (p > 0.05). After the induction of diabetes, differences in body weight of normal and diabetic rats were observed (p < 0.01). Both the extract and fraction of *Moringa stenopetala* leaves improved the weight gain compared with the diabetic control rats. By the end of the experiment, the body weight of the normal control group was significantly increased (p < 0.001). In contrast, the rats in the diabetic control group had slightly increased body weight during the experimental period (p < 0.05).

**Table 3:** Effects of aqueous ethanol and n-butanol fraction of *Moringa stenopetala* leaves on body weight in diabetic rats

	Body weight change during treatment (g)			
Treatment Groups	Before Induction	Day 0	Day 7	Day 14
Normal Control	221.60±5.43	262.43±4.95^a^	273.63±2.60^a^	280.83±2.08^a^
Diabetic Contro/DC/	211.03±3.46^b^	230.68±2.73^b^	229.58±6.11^b^	231.33±5.66^b^
DC + 500 mg/Kg ethanol extract of MS	212.52±2.96	233.65±4.21^b^	244.08±2.42^b^	250.57±2.43^ab^
DC + 500 mg/Kg butanol fraction of MS	212.70±3.12	230.03±2.87^b^	241.50±3.78^b^	249.67±5.55^b^
DC + I50 mg/Kg Metformin	220.32±5.41	236.67±6.04^b^	248.67±5.67^b^	249.50±6.31^b^

Results are expressed in Mean ± S.E.M, n = 6

^a^when p < 0.05 verses Diabetic control

^b^when p < 0.05 verses normal control

### Effect of the aqueous ethanol extract and n-butanol fraction of *Moringa stenopetala* leaves on lipid profiles

After the induction of diabetes and subsequent treatment with either the aqueous ethanol extract, the n-butanol fraction or metformin, there was a significant increase of serum total cholesterol, triglycerides, and significant decrease in HDL cholesterol in diabetic rats when compared to normal rats. The results showed that administration of aqueous ethanol extract and butanol fraction significantly decreased (P < 0.05) the levels of cholesterol and triglycerides (Table 4). HDL cholesterol level was also improved in diabetic rats after 14 days treatment (Table 4). In addition, metformin also improved the lipid profiles in diabetic rats.

**Table 4:** Effects of aqueous ethanol and n-butanol fraction of *Moringa stenopetala* leaves on lipid profiles in diabetic rats

Groups	TC (mg/dL)	TG (mg/dL)	HDL-C (mg/dL)	LDL-C(mg dL}
Normal control	84.0±1.7	81.5±1.7	29.3±1.8	43.76±2.7
Diabetic control/DC	189.5±2.3^a^	158.50±3.1^a^	19.8±0.2^a^	141.63±2.1^a^
DC + 500 mg/Kg ethanol extract of MS	127.5±1.1^ab^	93.25±3.7^ab^	25.54±1.4^ab^	83.11±1.3^ab^
DC + 500 mg/Kg butanol fraction of MS	97.2±1.5^b^	75.00±1.1^b^	28.67±1.5^b^	51.50±2.6^b^
DC + 150 mg/Kg Metformin	81.7±2.9^b^	64.67±1.5^b^	34.5±1.9^ab^	38.03±0.5^b^

The results are expressed Mean ± S.E.M (n = 6)

^a^p < 0.05 compared with normal control values

^b^p < 0.05 compared with diabetic control

### Effects of the aqueous ethanol extract and n-butanol fraction of *Moringa stenopetala* leaves on liver and kidney functions

The effects of *Moringa stenopetala* leaves on liver functions are shown in Table 5. The levels of ALT, AST, ALP, GGT and bilirubin were significantly elevated in diabetic rats. The rats treated with *Moringa stenopetala* extractions showed significant (P < 0.05) reduction in the elevated levels of liver enzymes (transaminase) and bilirubin as shown in Table 5. Diabetic rates showed a significant reduction in total protein levels whereas rats treated with the extraction of *Moringa stenopetala* leaves showed significantly increased levels (P < 0.05). The level of creatinine and urea were elevated in diabetic rats as compared with the normal rats (Table 6). Rats administered *Moringa stenopetala* extracts showed reduced levels of creatinine and urea compared to untreated diabetic rats (p < 0.05, Table 6). For the n-butanol fraction, the level of creatinine was not significantly different to that of non-diabetic rats (p > 0.05, Table 6). The results demonstrated that *Moringa stenopetala* reduced enzyme levels compared with diabetic control groups (Table 5).

**Table 5:** Effects of aqueous ethanol and n-butanol fraction of *Moringa stenopetala* leaves on liver function in diabetic rats

Groups	ALT (U/L)	AST (U/L)	ALP (U/L)	BLT(mg/dl)	GGT (U/L)
Normal control	32.0±2.2	57.8±2.35	53.01±0.8	0.13±0.01	3.55±0.26
Diabetic control/DC	73.0±1.7^a^	92.4±1.7^a^	85.66±1.24^a^	0.11 ±0.01	4.75±0.6
DC + 500 mg/Kg ethanol extract of MS	53.2±1,88^ab^	79.3±2.2^ab^	81.34±1.5^ab^	0.16±0.03	3.92±0.29
DC + 500 mg/Kg butanol fraction of MS	49.2±1.9^ab^	73.0±1.4^ab^	72.00±2.5^ab^	0.12± 0.04	3.67±0.27
DC + 150 mg/Kg Metformin	41.7±2.6^ab^	68.5±0.5^b^	61.23±1.4^ab^	0.10±0.01	3.82±0.37

The results are expressed Mean ± S.E.M (n = 6)

^a^p < 0.05 compared with normal control values

^b^p < 0.05 compared with diabetic control

**Table 6:** Effect of *Moringa stenopetala* leaves extract and its butanol fraction on urea, creatinine, and total albumin STZ induced diabetic rats

Groups	Urea (mg/dL)	Qreatdnme (mg/dL)	Total albumin (mg/dL.)
Normal control	40.2±1.6	0.85±0.13	6.1±0.12
Diabetic control/DC	79.0±1.5^a^	1.87±0.18^a^	4.57±0.11^a^
DC+500 mg/Kg ethanol extract of MS	56.5±1.8^ab^	1.35±0.23^ab^	5.23±0.13^b^
DC + 500 mg/Kg butanol fraction of MS	53.15±1.12^ab^	1.23±0.3^b^	5.81±0.13^b^
DC +150 mg/Kg Metformin	50.12±1.5^ab^	1.15±0.2^b^	5.79±0.11^b^

The results are expressed Mean ± S.E.M (n = 6)

^a^p < 0.05 compared with normal control values

^b^p < 0.05 compared with diabetic control

### Effects of aqueous ethanol extract and its butanol fraction of *Moringa stenopetala* leaves on histopathology of pancreas

**Fig. 1:**
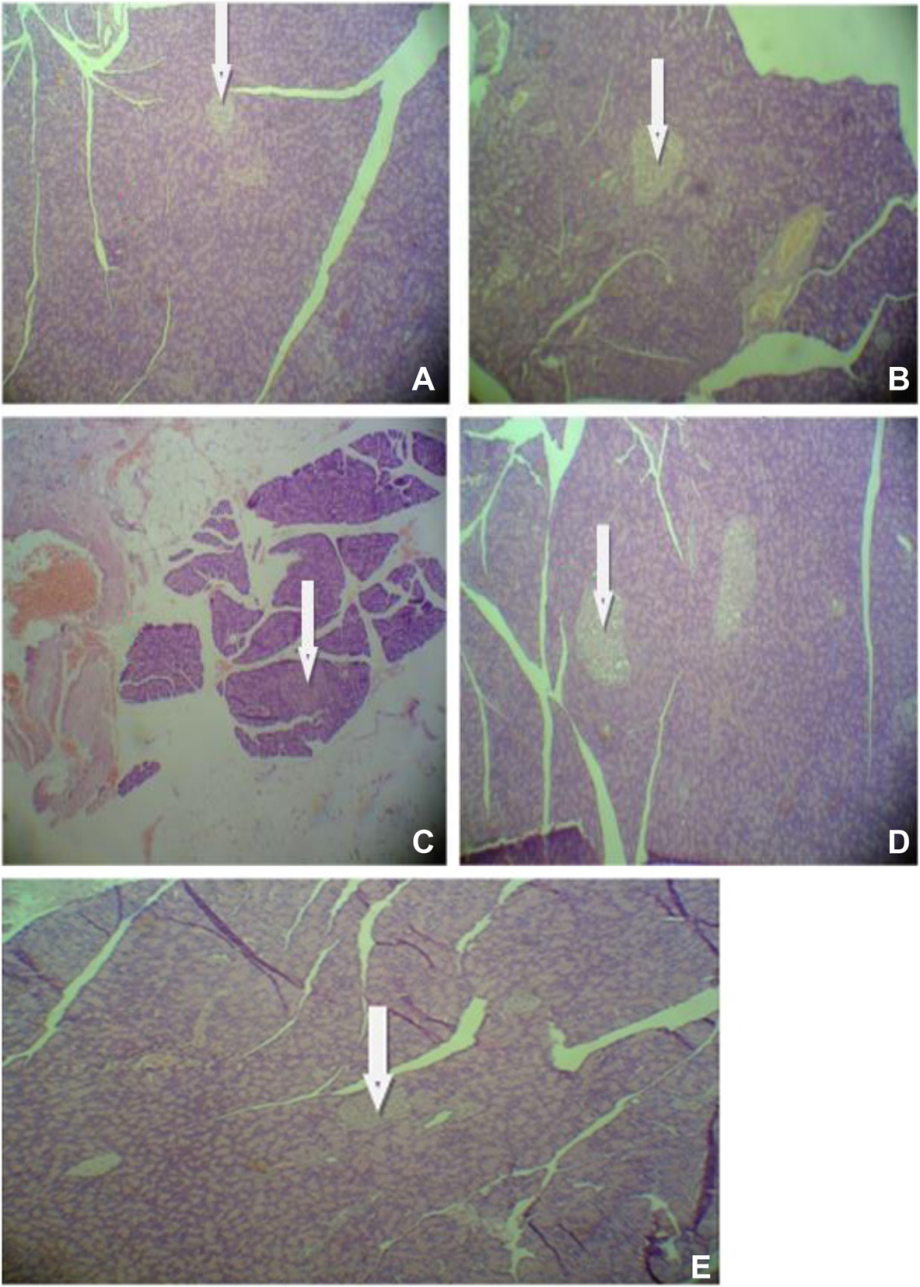
Histopathology of pancreas after administration of *Moringa stenopetala* in diabetic rats. **a**, x25: Pancreas of DC animal showing severe congestion of pancreatic parenchymal cells, infiltration of inflammatory cells and hyperplasia of islets cell. **b**, x25: Pancreas of diabetic animal treated with ethanol extract showing mild hyperplasia of islets cell and congestion of parenchymal cells. **c**, x25: Pancreas of diabetic animal treated with butanol fraction showing mild hyperplasia of islets cell and congestion of parenchymal cells. **d**, x25: Pancreas of NC animal showing normal histology. **e**, x25: Pancreas of diabetic animal treated with metformin showing reduced size of islets and devoid of fibrosis and necrosis

The structure of the pancreas of the normal control and diabetic rats are shown in Fig. 1. The pancreas of normal control rats showed normal islets, whereas that of diabetic rats showed hyperplasia of beta-cells and congestion of pancreatic parenchymal cells. Administration of the aqueous ethanol extract and n-butanol fraction of *Moringa stenopetala* leaves as well as metformin treatment increased the number of islets when compared to that of diabetic animals. The size of islets of Langerhans in the groups treated with aqueous ethanol and metformin was slightly reduced as compared to the normal control rats, however the group treated with n-butanol extraction shows islets of a similar size to that of the control group.

## Discussion

Diabetes mellitus is a metabolic disorder that usually affects carbohydrate, fat, and protein metabolism, followed by multiorgan dysfunctionin the later period, and hyperlipidemia associated with hyperglycemia [16]. Effective novel compounds with pan-target antidiabetic activity and proven long-term safety should be targeted in a clinical setting for patients with coexisting relevant lipid and glucose metabolic disorders. These discoveries pave the way for the development of drugs for treating chronic multigenic metabolic and cardiovascular diseases, for which therapy is presently insufficient or nonexistent [17, 18]. This is the first study to investigate the effect of aqueous ethanol and n-butanol fractions of *Moringa stenopetala* leaves on the regenerative behavior of pancreatic islets of Langerhans after streptozotocin induced damage of the pancreas and on biochemical effect after such treatment.

Streptozotocin (STZ) administration generally causes destruction of $\beta$-cells after three days in rats [19]. Pancreatic $\beta$-cells are particularly sensitive to damage by nitric oxide and free radicals because of the low levels of free radical scavenging enzymes in the tissue. The results of the present study indicate that the extracts of *Moringa stenopetala* produced regeneration/proliferation of the pancreatic$\beta$-cells possibly due to prevention of free radical formation induced by STZ. Since pancreas contains stable (quiescent) $\beta$-cells which have regenerative capacity, after damage the surviving cells proliferate by replication to supplicate the lost cells [20]. Regenerative pancreatic $\beta$-cells can be formed by neogenesis or by replication of the preexisting differentiated cells; since other medicinal plants have shown $\beta$-cell regenerative potential, it is possible that the extracts of *Moringa stenopetala* were also responsible for the proliferation of $\beta$-cells and the recovery of normal pancreatic morphology as shown in Fig. 1. Hence this implies that extracts of *Moringa stenopetala* were also responsible for the proliferation of $\beta$-cells as there are already reports showing extracts of other medicinal plants which have a $\beta$-cell regenerative potential [21]. This medicinal plant material has also shown neovascularization activity in the pancreatic islets of Langerhans and improved stromal fibrosis of pancreatic cells in histopathological investigations.

The presence of phytochemicals in plant products offers a great potential for balancing metabolic disturbances. Several phytomolecules, including flavonoids, total phenolic compounds, alkaloids, glycosides, saponins, glycolipids, dietary fibres, polysaccharides, peptidoglycans, carbohydrates, amino acids and others obtained from various plant sources, have been reported as potent hypoglycemic and antihyperglycemic agents. Flavonoids are a heterogeneous group of ubiquitous plant polyphenols, which exhibit a variety of pharmacological activities, including the anti-atherogenic as well as antihyperglycemic effects, lipoprotein oxidation, blood platelet aggregation and vascular reactivity [22, 23]. A high content of phytochemicals, especially total polyphenolic compounds and total flavonoids, may contribute to the pleiotropic effects of *Moringa stenopetala* leaves that support the use of the plant for different metabolic disorders in the local community [14]. A high content of total phenolic compounds and flavonoids may have a significant role in regulating metabolic disturbances which are highly related to diabetes mellitus and its complications due to its protective effect on the pancreas and other essential organs.

The most common lipid abnormalities in diabetes are hypertriglyceridemia and hypercholesterolemia [24]. Repeated administration of *Moringa stenopetala* leaves extract and fraction for 14 days significantly decreased hypertriglyceridemia and hypercholesterolemia. The observed antihypelipidemic effect may be due to decreased cholesterogenesis and fatty acid synthesis through inhibition of pancreatic cholesterol esterase and pancreatic lipase inhibition effect, respectively [25, 26]. The HDL cholesterol level was significantly improved by *Moringa stenopetala* leaf extract treatment. In our previous reports we have shown that this plant material had inhibitory effects on pancreatic cholesterol esterase and pancreatic lipase [14] which may contribute to antihyperlipidemic activities in animal models.

Enzymes indicating liver damage, such as AST, GGT, ALT and ALP level increased in diabetic rats. The elevated serum level of these enzymes was significantly reduced by *Moringa stenopetala* treatment. There is evidence that the diabetic complications such as increased gluconeogenesis and ketogenesis may be due to elevated enzymes [27]. The restoration of transaminases to their normal levels after treatment indicates revival of insulin secretion and regenerative activities of islets of Langerhans cells of pancreas after administration of the plant material. *Moringa stenopetala* also improved renal functions in diabetic rats by reducing serum urea and creatinine levels. Therefore, our results demonstrate that *Moringa stenopetala* is able to normalize vital organs function in rats.

Streptozotocin has been shown to induce free radical production and cause tissue injury. Previous studies on histopathological investigation of organs such as liver and kidney have shown that *Moringa stenopetala* was non-toxic [28]. The pancreas is especially susceptible to the action of streptozotocin induced free radical damage. *Moringa stenopetala* leaves are rich in total polyphenolic compounds and flavonoid and have an *in vitro* antioxidant effect [13, 14]. This antioxidant activity of the plant may protect the organ from free radicals. Various studies have shown that diabetes is associated with increasing formation of free radicals and decreasing antioxidant potential [29]. The effect of plant material as renoprotective, hepatoprotective and pancreatoprotective parameters may be highly associated with its antioxidant activity.

## Conclusion

These findings demonstrated that aqueous extract of *Moringa stenopetala* leaves and its butanol fraction possess antihyperglycemicand antihyperlipidemic properties, and alleviates streptozotocin induced pancreatic damage. Thus plant material of *Moringa stenopetala* could be used for prevention/treatment of hyperglycemia and hyperlipidemia. The beneficial effects of plant material in inhibition of diabetes induced complications such as protein glycation are being investigated. Further studies are warranted to investigate how the plant material normalize blood glucose and lipid levels, and whether insulin secretion or sensitization occur up on administration of Moringa stenopetala leaves extract.
